# Sleep and Mental Development

**Published:** 1906-01-27

**Authors:** 


### SLEEP AND MENTAL DEVELOPMENT.

A discussion has for some time been proceeding in the non-medical press, the full importance of which has been to a great extent obscured by the urgency of political strife during the preparations for and the conduct of the general election. This discussion has had reference to the question whether the time allowed for uninterrupted sleep, in many schools, is really sufficient for the needs of growing children; and it was originally started by a memorial from the Association of Medical Officers of Schools, founded upon a paper read before that body by Dr. Acland. The continuance of the discussion has served to bring into curious prominence the failure of large numbers of persons, and, among them, of many eminent members of the teaching profession, to realise that, in the present state of knowledge, no severance between "bodily" and "mental" acts or activities is possible. It has been gravely maintained that "mental" action is something different from, and even distinguishable from, "bodily" action; and it has been more than suggested that the energetic performance of the latter will in some way afford relief from fatigue incidental to the former.

The question of what is meant by "mind," of what may be its nature, and of how far it can be supposed to exist as an independent entity, we are content to leave to metaphysicians; and we approach the question only as physiologists. From this point of view, the intellectual acts of a schoolboy, the recollection of grammatical rules, or of the meanings of words in classic tongues, are not acts of the "mind," but acts of the *brain;* and the brain, like other structures of the body, is susceptible of exhaustion from over-work, and requires constant renewal of its energies by the action of sleep and food. In relation to these requirements, it is not possible to distinguish the divisions of the nervous system which govern muscular from those which govern intellectual effort; and to maintain, as is apparently maintained by many schoolmasters, that bodily exercise is itself recuperative after "mental" effort, so that a boy who has been exhausted by study can be restored to his pristine "mental" vigour by a paper chase or by compulsory running, or by any other form of severe and sustained "bodily" exertion, seems to us to be the very extremity of physiological ignorance and of practical unwisdom. It is like lighting a candle at its lower end to make amends for its consumption at the top. The real question is simply whether the sum total of the boy's efforts, cerebral and muscular, are or are not in excess of his power to repair damages as fast as they are occasioned, and, at the same time, to provide for the proper growth of his muscles and for the proper development of his brain-cells. For the fulfilment of these essential steps towards the maturation of his powers, whether muscular or intellectual, a sufficiency of sleep is one of Nature's conditions; and the inquiry whether the amount of sleep actually obtained, in any given case, is sufficient for the wants of the individual, is one for the physician or for the physiologist, and not at all for the members of the teaching profession. We have known more than one instance in which a boy, distinguished at his school, has employed the early days of his holidays chiefly in making up the arrears of sleep which had become due to him during the term, and in which only the opportunities of doing so had stood between him and complete breakdown. The time has come for schoolmasters to recognise that we know nothing about "mind," and that "brain" is in no way an exception to other bodily organs.

